# Lysosome-targeting agents in cancer therapy

**DOI:** 10.18632/oncotarget.21451

**Published:** 2017-10-03

**Authors:** Guido Kroemer, Lorenzo Galluzzi

**Affiliations:** ^1^ Université Paris Descartes/Paris V, Paris, France; ^2^ Université Pierre et Marie Curie/Paris VI, Paris, France; ^3^ Equipe 11 labellisée Ligue contre le Cancer, Centre de Recherche des Cordeliers, Paris, France; ^4^ INSERM, U1138, Paris, France; ^5^ Metabolomics and Cell Biology Platforms, Gustave Roussy Comprehensive Cancer Institute, Villejuif, France; ^6^ Department of Women’s and Children’s Health, Karolinska Institute, Karolinska University Hospital, Stockholm, Sweden; ^7^ Pôle de Biologie, Hopitâl Européen George Pompidou, AP-HP, Paris, France; ^8^ Department of Radiation Oncology, Weill Cornell Medical College, New York, NY, USA; ^9^ Sandra and Edward Meyer Cancer Center, New York, NY, USA

**Keywords:** anticancer immunosurveillance, autophagy, chemotherapy, chloroquine, hydroxychloroquine

## Abstract

Despite considerable efforts from multiple laboratories worldwide, highly specific inhibitors of autophagy for clinical use are not yet available. Lysosomal inhibitors are being employed instead, in spite of multiple limitations that are summarized herein.

Levy and colleagues have recently provided a comprehensive and balanced overview on the therapeutic potential of autophagy-targeting agents for cancer therapy [1]. Indeed, owing to its privileged position at the hub of multiple, if not all, cellular processes [2], autophagy stands out as a promising target for the development of new therapeutic regimens against a large panel of human disorders [3]. Unfortunately, truly specific pharmacological inhibitors of autophagy are not yet available for use in cancer patients [3].

Chloroquine and hydroxychloroquine have been extensively tested as antineoplastic agents in preclinical and clinical settings with promising results [4, 5], but these antimalarial drugs operate by inhibiting lysosomal acidification, which affects autophagy as well as all forms of vesicular trafficking [6]. In line with this notion, chloroquine and hydroxychloroquine have been shown to inhibit the growth of malignant cells *in vitro* and *in vivo* by autophagy-independent mechanisms [7–9].

Another potential issue with using chloroquine and hydroxychloroquine for oncological indications is linked to their immunosuppressive effects [10]. Indeed, it is now widely accepted that the elicitation of antitumor immunity is key to long-term therapeutic efficacy in patients affected by most, if not all, malignancies [11]. In this setting, the systemic administration of lysosomal inhibitors such as chloroquine and hydroxychloroquine might therefore be detrimental as it would favor the escape of malignant cells from immunosurveillance [12]. Accordingly, the administration of chloroquine to 39 patients with brain metastases from solid tumors receiving whole-brain irradiation (WBI) improved locoregional disease control (as compared to 39 patients who received WBI only), but failed to ameliorate response rate and overall survival [13]. Thus, at least in some settings, combining standard-of-care therapeutic regimens with lysosomal inhibitors may confer short-term benefits that are not paralleled by an actual extension in patient survival. Finally, although results from multiple clinical trials indicate that these agents are well tolerated by patients with a variety of neoplasm [5], experience from other fields of investigation suggests that the toxicities of chloroquine and hydroxychloroquine must be taken into attentive consideration [10].

The development of truly specific pharmacological inhibitors of autophagy for clinical use will shed additional light on the actual therapeutic potential of this approach for the management of neoplastic conditions. We surmise that refined targeted strategies will have to be conceived to focus the therapeutic effects of autophagy inhibitors on cancer cells as anticancer immune responses (which also rely on autophagy) remain fully operational.
